# Molecular-Marker-Based Design for Breeding Indica–Japonica Hybrid Rice with Bacterial Blight Resistance

**DOI:** 10.3390/genes16060719

**Published:** 2025-06-18

**Authors:** Junjie Dong, Xinyue Zhang, Youfa Li, Haowei Fu

**Affiliations:** Jiaxing Academy of Agricultural Sciences, Jiaxing 314010, China; dongjunjie0623@163.com (J.D.); drzhangxinyue@163.com (X.Z.); liyoufa66@sina.com (Y.L.)

**Keywords:** bacterial blight, wide compatibility, molecular-marker-assisted selection

## Abstract

**Background/Objectives**: To overcome the limitations imposed by bacterial blight on widely adopted indica–japonica hybrid rice, this study employed molecular design breeding strategies to develop a resistant germplasm. **Methods**: Through conventional backcross breeding combined with molecular-marker-assisted selection, the *Xa23*-carrying material XR39 was hybridized with the wide-compatibility restorer line R5315 harboring the *S5n* gene. Progeny selection integrated evaluations of agronomic traits, disease resistance identification, and test-crossing with sterile lines. **Results:** Five wide-compatibility restorer lines simultaneously incorporating the *Xa23* and *S5n* genes were successfully developed, demonstrating outstanding bacterial blight resistance and restoration ability. The selected hybrid combinations, A3/RP1, A1/RP4, and A4/RP4, exhibited yield increases of 2.6–8.6% compared to the control. **Conclusions**: This study not only established a novel germplasm for developing bacterial blight-resistant indica–japonica hybrid rice varieties, but also established a model for gene design breeding for rice improvement.

## 1. Introduction

Bacterial blight caused by *Xanthomonas oryzae* is one of the most destructive diseases in rice [1]. In epidemic years, production in the affected areas is generally reduced by 20%–30%, reaching up to 80% in severe cases [2,3]. In recent years, the incidence of bacterial blight in Zhejiang Province, China, has been increasing. Between 2014 and 2016, late rice cultivation in Zhejiang Province experienced numerous disease outbreaks during the later stages of growth [4,5]. These outbreaks were observed in various regions including Wenzhou, Taizhou, Ningbo, Shaoxing, Jinhua, Lishui, and Quzhou, indicating a rapid dissemination in certain areas. In 2019, the late-rice-affected area in Zhejiang Province was 16,700 hectares, which increased to over 32,700 hectares in 2021. Particularly severe damage has been observed in specific regions such as Taizhou, Jinhua, and Quzhou, leading to persistent late rice crop failures. The situation regarding prevention and control is notably critical. Developing and promoting rice varieties with multiple resistances is the most effective approach for disease and pest prevention and management [6]. Forty-two genes providing resistance to rice bacterial blight have been identified to date [7,8]. Among them, the *Xa23* gene, known for its broad-spectrum resistance, high resistance levels, and durable resistance properties, was originally identified in common wild rice by Wang et al. [9], and created a nearly isogenic line CBB23, which is widely used in breeding. Wang et al. [10,11] initially mapped the *Xa23* locus to a 49.8 kb region on chromosome 11, and then further cloning studies showed that the susceptible *Xa23* allele has an identical open reading frame of *Xa23* but differs in the promoter region for lack of the TALE binding element for *AvrXa23.* Tian et al. [12] demonstrated that *Xa23* maintained its broad-spectrum resistance to the Xoo strain in rice cultivation regions of southern China between 2019 and 2021.

In 1987, Yuan Longping introduced three stages of hybrid rice breeding incorporating intervariety, intersubspecies, and distant hybridization advantages [13]. Indica–japonica hybrid rice exhibits traits such as large panicles, high grain count, sturdy stems, robust growth, and well-developed root systems. However, the practical application of intersubspecific heterosis has been significantly hindered by natural postzygotic reproductive isolation between indica and japonica subspecies, manifesting as hybrid sterility and low seed setting rates in offspring [14]. The *S5n* gene locus, which regulates female gamete fertility in rice, was initially identified by Ikehashi on chromosome 6 [15]. Subsequent research has focused on the precise localization and exploration of the *S5n* locus [16,17]. Through map-based cloning and genetic analysis, it has been established that the three linked genes (ORF3, ORF4, ORF5) at the *S5n* locus interact genetically, constituting a “killer–protector” system that governs the fertility and segregation of female gametes in rice indica–japonica hybrids [18]. A critical breakthrough came with the identification of a 136 bp deletion in *S5n* alleles differentiating indica and japonica subspecies [16], which enabled the development of the functional InDel marker S5-136 for molecular screening [19]. This genetic marker has since become an essential tool for marker-assisted selection breeding and germplasm characterization [20,21]. In China’s Zhejiang Province, these scientific advances have facilitated the successful development of three-line indica–japonica hybrid rice systems. Notable commercial varieties, including Yongyou, Zheyou, Chunyou, and Jiayou series, combine japonica cytoplasmic male sterile lines with wide-compatibility indica restorer lines. These achievements demonstrate the effective translation of intersubspecific hybridization research into practical agricultural applications through the strategic integration of germplasm resources and molecular breeding technologies.

In this study, the rice intermediate material XR39, harboring the *Xa23* gene for bacterial blight resistance, was crossed with R5315, a wide-compatibility restorer line carrying the *S5n* gene. Through molecular-marker-assisted selection, five wide-compatibility restorer lines with the *Xa23* gene were identified. The indica–japonica hybrid rice derived from this combination exhibits strong resistance to bacterial blight, high yield potential, and promising developmental prospects.

## 2. Materials and Methods

### 2.1. Experimental Materials

XR39 and R5315 were provided by Jiaxing Academy of Agricultural Sciences. Four different BT-type japonica rice sterile lines (A1–A4) were used, namely, Jiatong A, Jianing A, Jia238A, Quanjing 1A, Jiatong A, Jianing A, and Jia238A, which were independently selected by Jiaxing Academy of Agricultural Sciences, while Quanjing 1A was provided by Anhui Quanyin High tech Seed Industry Co., Ltd., Hefei, China.

### 2.2. RP1–5 Breeding Process (Figure 1)

#### 2.2.1. Parental Material Development (2019–2020)

Summer 2019: Hybridization was conducted at the experimental field of Jiaxing Academy of Agricultural Sciences using the bacterial-blight-resistant material XR39 (carrying the *Xa23* resistance gene) as the maternal parent and the wide-compatibility restorer line R5315 as the paternal parent, yielding 15 hybrid seeds.

Spring 2020: F1 generation screening was performed at the Lingshui experimental station in Hainan. False hybrids were removed through phenotypic identification, and 13 true hybrid plants were retained and bulk-harvested.

#### 2.2.2. Population Construction and Multi-Generational Selection (2020–2022)

Summer 2020: Approximately 4000 F2 plants were cultivated at Jiaxing. Bacterial blight resistance was evaluated at the booting stage, and 45 elite plants were selected based on comprehensive agronomic traits at maturity. Molecular-marker-assisted selection (MAS) further narrowed the pool to 32 plants.

Spring 2021: A total of 34 elite plants from 12 F3 lines were retained at the Lingshui station after MAS and agronomic trait evaluation.

Summer 2021: Through continuous self-pollination and field selection, 30 stable F4 lines from 10 families were obtained in Jiaxing.

Spring 2022: Molecular marker validation of 30 F5 lines was conducted in Hainan. Concurrently, test crosses were performed with male sterile lines (e.g., Jianing A, Jiatong A), yielding 90 hybrid combinations.

#### 2.2.3. Combining Ability Evaluation (2022–2023)

Summer 2022: At Jiaxing, combining ability tests identified 5 superior hybrid combinations (RP1–RP5). Three plants per combination were bulk-harvested, totaling 15 core lines.

Spring 2023: Expanded test crosses with four male sterile lines (Jianing A, Jiatong A, Jia238 A, Quangeng1 A) generated 120 new hybrid combinations.

#### 2.2.4. Line Stabilization and Field Trials (2023–2024)

Summer 2023: A re-evaluation of 120 F8 combinations at Jiaxing confirmed the superiority of RP1–RP5.

Spring 2024: Small-scale seed production was initiated in Hainan, generating 20 hybrid combinations with four male sterile lines.

Summer 2024: Field trials and community demonstrations at Jiaxing evaluated the agronomic performance, disease resistance, and yield potential of the new combinations.

**Figure 1:**
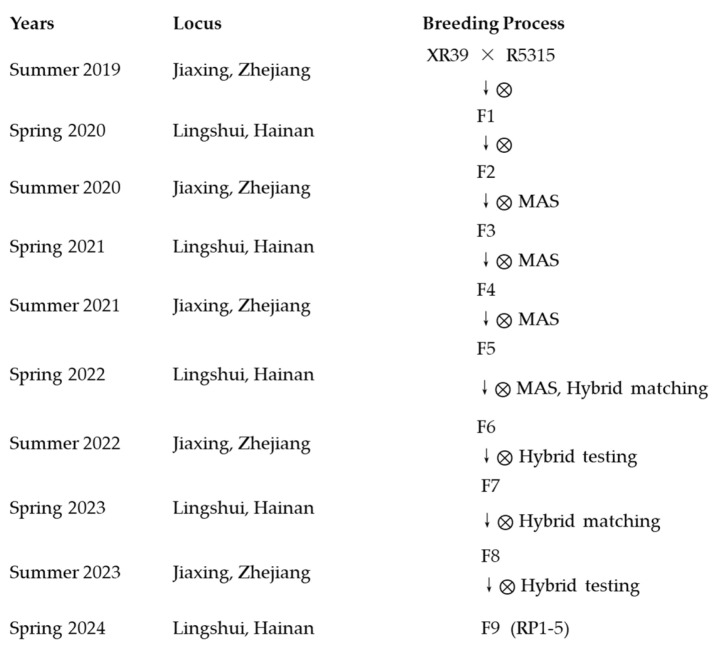
Selection process for stable strains with bacteria blight resistance.

### 2.3. Management of Experimental Fields and Evaluation of Agronomic Traits

The experimental design employed a systematic planting configuration with 6 rows of 6 plants per line (36 plants per line) for each generation, and each hybrid combination was cultivated in 45 rows containing 6 plants per row at a spacing of 15 cm between rows and 20 cm within rows. Yongyou 1540, an indica–japonica hybrid rice variety from the regional trial in China, was used as the control.

The heading stage of rice was recorded. After the rice matured, the edge rows were removed from in each plot. Three plants were selected from the middle rows, and the plant height (PH), effective panicle number per plant (EPNPP), thousand-grain weight (TGW), total grain number per panicle (TGNPP), seed setting rate (SSR), single panicle weight (SPW), grain length (GL), and grain width (GW) were measured.

### 2.4. Molecular Marker Detection

DNA was extracted from leaf tissue using a rapid method reported by Zeng et al. [22], with minor modifications. The bacterial blight resistance gene *Xa23* was identified using the linked marker RM26985 and the functional marker Xa23-fun [23]. The wide-compatibility gene *S5n* was detected using marker S5136 [19]. The primers used were synthesized by Shanghai Sangon Biotech Co., Ltd., Shanghai, China and specific sequence information is detailed in Table 1. The PCR reaction mixture (20 μL total volume) contained 10 μL 2 × PCR Mix, 1 μL each of 10 μmol/L forward and reverse primers, 2 μL DNA template, and 6 μL ddH_2_O. The PCR program was as follows: 94 °C for 5 min; 29 cycles of 94 °C for 1 min, 55 °C for 1 min, 72 °C for 1 min, and a final extension at 72 °C for 7 min. Amplified products were separated using 4% agarose gel electrophoresis and visualized using nucleic acid dye.

### 2.5. Identification of Bacterial Blight Resistance in the Field

The identification strain P6 was provided by the Institute of Plant Protection and Microbiology, Zhejiang Academy of Agricultural Sciences. The artificial leaf cutting method was employed to evaluate the rice bacterial blight resistance at the heading stage. This involved dipping scissors into a bacterial solution and cutting approximately 2 cm of sword leaves, with 4–5 leaves being cut from each plant. Disease incidence was assessed 20 days post-inoculation following the criteria established by Jiang et al. [24] (Table 1).

### 2.6. Statistical Analyses

Data organization and statistical analysis were conducted using Microsoft Excel 2010, GraphPad Prism 8, and SAS 9.4. One-way ANOVA was used to analyze variance and *t*-tests were conducted with significance levels of *p* < 0.05 and *p* < 0.01.

## 3. Results

### 3.1. Molecular Detection of Resistance Gene Xa23 and Wide-Compatibility Gene S5n in F2 Populations

**Figure 2:**
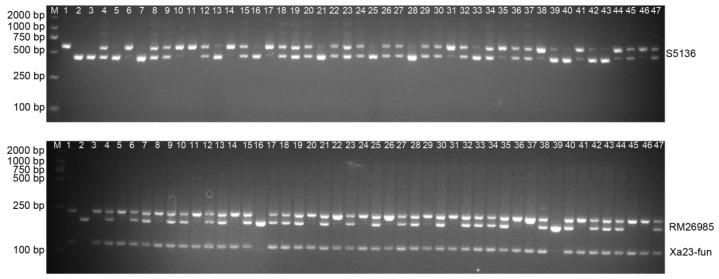
Molecular detection of resistance gene *Xa23* and wide-compatibility gene *S5n* in F2 populations (M: DL2000 DNA Marker; 1: XR39; 2: XR5315; 3–47: F2 populations).

The analysis employed the linkage marker RM26985 and the functional marker Xa23-fun for *Xa23* identification, along with the linkage marker S5136 for *S5n* detection (Table 2). Distinct amplification patterns were observed: RM26985 produced 183 bp and 166 bp fragments in XR39 and XR5315, respectively; Xa23-fun generated a 105 bp fragment in XR39, but no effective product in XR5315; and S5136 amplified 577 bp and 441 bp fragments in XR39 and XR5315, respectively (Figure 2). Notably, two materials lacking the *Xa23* gene were identified through molecular marker analysis, despite prior phenotypic selection of resistant plants following artificial inoculation with the bacterial blight pathogen *Xanthomonas oryzae*. This discrepancy indicates that phenotypic selection alone may yield false positives due to a measurable error rate in resistance identification. Significant polymorphism was observed in these markers, both between parental lines XR39 and XR5315 and within the F2 segregating populations. These findings validate the effectiveness of these markers for implementing marker-assisted selection in hybrid breeding programs.

### 3.2. Breeding of Wide-Compatibility Restorer Lines Resistant to Bacterial Blight

**Figure 3:**
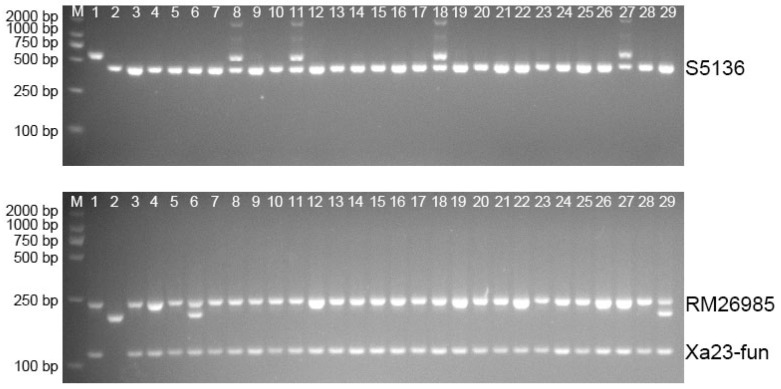
Molecular detection of resistance gene *Xa23* and wide-compatibility gene *S5n* in F6 populations (M: DL2000 DNA Marker; 1: XR39; 2: XR5315; 3–29: F6 populations).

The parental line XR39 containing the *Xa23* gene was crossed with the parental line R5315 harboring the wide-compatibility gene *S5n*. Molecular markers were employed to screen the progeny plants, and the results were combined with field inoculation identification until a homozygous disease-resistant line was obtained. Figure 3 presents the molecular detection results of some materials from the high-generation population. It can be observed that all of the F6 generation carries both the Xa23 gene for resistance to bacterial blight and the *S5n* gene for wide compatibility.

### 3.3. Bacterial Blight Resistance Performance of Five Wide-Compatibility Restorer Lines and Twenty Hybrid Combinations

**Figure 4:**
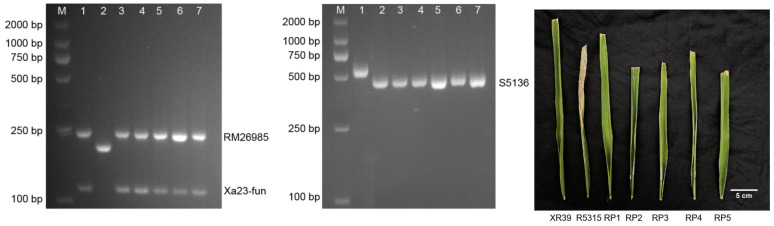
Molecular detection of resistance gene *Xa23* and wide-compatibility gene *S5n* of five wide-compatibility restorer lines (M: DL2000 DNA Marker; 1: XR39; 2: XR5315; 3–7: RP1, RP2, RP3, RP4, RP5).

After agronomic trait evaluation and combining ability testing, five wide-compatibility restorer lines (RP1–5) carrying the *Xa23* and *S5n* genes for resistance to bacterial blight were established in the F8 generation (Figure 4). Bacterial blight resistance was examined in five wide-compatibility restorers and their 20 hybrid combinations using Xanthomonas oryzae P6. The results show that, except for R5315 and Yongyou 1540, which were not detected in Xa23, all materials were resistant to bacterial blight at the boot stage (Table 3).

### 3.4. Main Agronomic Trait Performance of Five Wide-Compatibility Restorer Lines and Twenty Hybrid Combinations

According to Table 4, RP1 and RP5 had the shortest sowing duration, both 4 days earlier than R5315. The plant height of RP2, RP3, RP4, and RP5 was significantly lower than that of R5313, suggesting that their lodging resistance may be improved. The analysis of yield-related agronomic traits showed that the effective panicle number per plant of the five wide-compatibility restorer lines were not significantly different from R5315. The single panicle weight of RP1 and RP4 was significantly higher than that of R5315. RP1 benefited from significantly higher total grain number per panicle and seed setting rate than R5315. RP4 benefited from significantly higher 1000-grain weight and total grain number per panicle than R5315. The analysis of grain type traits showed that RP2 had the longest grain and RP1 had the shortest grain, which were 8.75 mm and 7.66 mm, respectively. RP4 has the narrowest grain and RP2 has the widest grain at 2.29 mm and 2.79 mm, respectively. Considering grain-related traits, RP2, a large grain restorer line, has the highest 1000-grain weight and the widest and longest grains. RP4 grains are a slender grain type and exhibit the largest length/width ratio.

**Figure 5:**
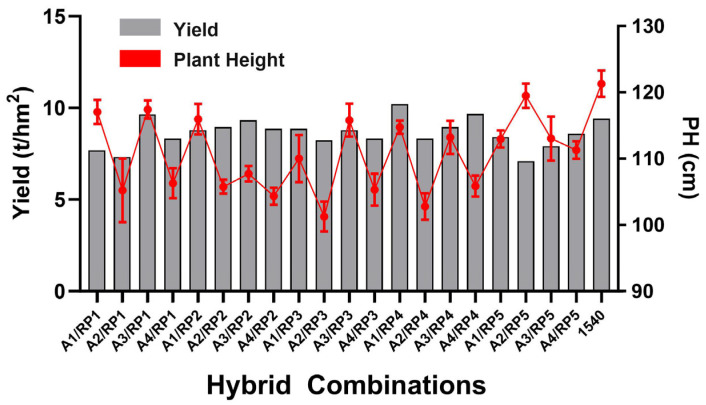
Yield and plant height of hybrid combinations.

The agronomic characteristics of 20 hybrid combinations showed that the yields of A3/RP1, A1/RP4, and A4/RP4 were higher than those of the control, Yongyou 1540, with an increase of 2.6%, 8.6%, and 2.9%, respectively (Table 5). Further, the total grain number per panicle of the combinations A3/RP1 and A4/RP4 was higher than that of the control, while the combination A1/RP4 had a higher total grain number per panicle and 1000-grain weight than that of the control. The duration from seeding to heading of the three high-yielding combinations was earlier than that of the control, and the plant height was lower. Among them, the duration of A4/RP4 was 21 days earlier than that of the control, and the plant height was 13.2% lower; A3/IRP1 was 7 days earlier than the control, and the plant height was 3.2% lower; and A1/RP4 was 7 days earlier than the control, and the plant height was 5.4% lower. The three combinations have the characteristics of short plant height, early growth period, and high yield, indicating that they have good prospects for production and application (Figure 5).

## 4. Discussion

Recently, rice bacteria blight has become severe in some regions of China, posing significant risks to rice safety production. By improving the defense system of rice itself, the cultivation of a new variety of long-spectrum disease-resistant rice is the most environmentally friendly and most effective disease prevention measure [25]. Molecular-marker-assisted selective breeding can be carried out at the genetic level, which is one of the best means to achieve the high efficiency polymerization of multiple excellent traits. The *Xa23* gene is an apparent rice bacterial blight resistance gene, which has been widely used in the bacterial blight resistance of hybrid rice. Huang et al. [26] successfully introduced the *Xa23* gene into the two-series infertility line Quan 211S with the use of marker-assisted selection technology. Yang et al. [27] used the full genome background to choose technology to introduce the *Xa23* gene into the two-series infertility line Feng 39S. The genetic background was successfully selected in a similar way to Feng 39S. Huang et al. [28] found that the conventional japonica rice Ning 84 imported the *Xa23* gene using marker-assisted selection technology, significantly improving the resistance to bacterial leaf blight. In previous studies, the majority of *Xa23* gene applications used indica hybrid rice, with few involving indica–japonica hybrid rice [26,27]. Thanks to recent genetic research and the selection of recovery systems, indica–japonica hybrid rice has been promoted and applied in China [29]. Song et al. [30] selected the *S5n* gene through molecular-marker-assisted selection to breed the broad affinity restorer line Zhehui 810, and further developed a new indica–japonica hybrid rice variety Zheyou 810. In this study, *Xa23* was detected through the linked marker RM26985 in the low generation, and the functional marker Xa23-fun with complete dominance was used in the high generation. The results show that inoculation and appraisal are highly consistent. There are multiple reasons for the wide-compatibility molecular mechanism in rice, and its genetic regulation is regulated in many different ways [14]. Our study used the marker S5136 linked with *S5n* to detect the wide compatibility. Combined with the determination of coordination force, five wide-compatibility restorer lines were successfully bred, further verifying the applicability of the molecular marker S5136. Further, we tested the broad compatibility restorer line with resistance to bacterial blight selected in this study and matched it with four three-line japonica CMS lines. Among them, the three combinations A3/RP1, A1/RP4, and A4/RP4 have excellent agronomic traits, indicating good development prospects. Accordingly, the research ideas and methods are feasible for developing new indica–japonica hybrid rice varieties by breeding wide-compatibility restorer lines with resistance to bacterial blight by aggregating *S5n* and *Xa23* genes.

## 5. Conclusions

The bacterial blight-resistant indica–japonica hybrid rice combinations developed in this study exhibit robust disease resistance, high yield potential, and favorable agronomic traits, providing novel breeding materials with dual disease resistance and wide compatibility for ongoing improvement. By adopting a marker-assisted gene pyramiding strategy, this study achieved the technical integration of disease resistance resources and distant hybridization advantages, offering a paradigm for breeding new rice varieties.

## Figures and Tables

**Table 1:** Scoring of bacterial blight resistance.

Lesion Rating	Resistance Level	Lesion Length
0	HR	<1 cm
1	R	1–3 cm
3	MR	<1/4 of the inoculated leaf length
5	MS	1/4–1/2 of the inoculated leaf length
7	S	1/2–3/4 of the inoculated leaf length
9	HS	>3/4 of the inoculated leaf length

**Table 2:** Molecular marker information for identification.

Gene	Primer	Primer Sequence (5′-3′)	Fragment Length (bp)
*Xa23*	RM26985-F	CACAAGACAACCTTCAATGG	183/166
	RM26985-R	GGCTTAGGAGCGTTTATAGG	
	Xa23-fun-F	AAAGTCCCTTCCGAAACATC	105/-
	Xa23-fun-R	ATGAGGAAGTGCTGCCAGA	
*S5n*	S5136-F	ATCAACCCATTTCCTTTCCT	577/441
	S5136-R	ATACGCTCGATCGGATTAAC	

**Table 3:** Identification of resistance to bacterial blight in five wide-compatibility restorers and their hybrid combinations.

Material	Lesion Rating	Resistance Level	Material	Lesion Rating	Resistance Level
XR39	0	HR	A4/RP2	1	R
R5315	9	S	A1/RP3	0	R
RP1	0	HR	A2/RP3	1	R
RP2	0	HR	A3/RP3	1	R
RP3	0	HR	A4/RP3	1	R
RP4	0	HR	A1/RP4	0	R
RP5	0	HR	A2/RP4	1	R
A1/RP1	0	HR	A3/RP4	1	R
A2/RP1	1	R	A4/RP4	1	R
A3/RP1	1	R	A1/RP5	0	R
A4/RP1	1	R	A2/RP5	1	R
A1/RP2	0	R	A3/RP5	1	R
A2/RP2	1	R	A4/RP5	1	R
A3/RP2	1	R	1540	9	S

**Table 4:** Agronomic characteristics of wide-compatibility restorer lines with bacterial blight resistance.

Traits	RP1	RP2	RP3	RP4	RP5	R5315
DSH (d)	77.00	81.00	81.00	80.00	77.00	81.00
PH (cm)	103.4 ± 1.4 a	95.0 ± 1.8 c	96.5 ± 1.2 bc	96.5 ± 1.3 bc	97.6 ± 1.2 b	104.8 ± 1.3 a
EPNPP	7.4 ± 0.5 a	7.8 ± 0.8 a	8.0 ± 1.0 a	8.0 ± 0.7 a	8.2 ± 0.8 a	7.2 ± 0.8 a
TGW (g)	19.3 ± 0.2 e	22.3 ± 0.3 a	20.9 ± 0.4 c	21.4 ± 0.2 b	20.3 ± 0.2 d	20.1 ± 0.3 d
TGNPP	378.1 ± 3.0 a	217.9 ± 1.5 e	251.7 ± 3.8 d	344.7 ± 5.6 b	303.4 ± 5.5 c	311.5 ± 6.4 c
SSR (%)	85.2 ± 0.6 a	80.6 ± 1.8 b	76.9 ± 1.9 c	79.1 ± 0.9 bc	77.3 ± 1.2 c	81.3 ± 0.3 b
SPW (g)	6.2 ± 0.1 a	3.9 ± 0.1 e	4.0 ± 0.1 e	5.8 ± 0.1 b	4.8 ± 0.1 d	5.1 ± 0.2 c
GL (mm)	7.66 ± 0.14 d	8.75 ± 0.07 a	8.18 ± 0.09 b	8.17 ± 0.04 b	7.93 ± 0.04 c	8.28 ± 0.07 b
GW (mm)	2.48 ± 0.04 b	2.79 ± 0.03 a	2.42 ± 0.03 c	2.29 ± 0.03 d	2.41 ± 0.02 c	2.42 ± 0.01 c
GL/GW	3.09 ± 0.01 f	3.13 ± 0.01 e	3.39 ± 0.01 c	3.56 ± 0.02 a	3.29 ± 0.01 d	3.42 ± 0.01 b

The data are presented as means ± standard deviation (SD); different letters indicate significant difference at the *p* < 0.05 level. DSH: Duration from seeding to heading; PH: plant height; EPNPP: effective panicle number per plant; TGW: 1000-grain weight; TGNPP: total grain number per panicles; SSR: seed setting rate; SPW: single panicle weight; GL: grain length; GW: grain width.

**Table 5:** Agronomic characteristics of combinations between five wide-compatibility restorer lines with bacterial blight resistance and four japonica CMS lines.

Hybrid Combinations	DSH (d)	PH (cm)	EPNPP	TGW (g)	TGNPP	SSR (%)	SPW (g)	Yield (t/hm^2^)	GL (mm)	GW (mm)	GL/GW
A1/RP1	97.0	117.0 ± 1.8 **	9.2 ± 0.8	18.9 ± 0.1 **	300.2 ± 5.0 **	65.2 ± 0.9 **	3.7 ± 0.1 **	7.68	7.68	2.68	2.89
A2/RP1	101.0	105.2 ± 4.8 **	8.8 ± 0.8	20.6 ± 0.2	369.7 ± 4.5 **	74.6 ± 1.1 **	5.7 ± 0.1 *	7.32	7.75	2.67	2.92
A3/RP1	98.0	117.4 ± 1.4 **	9.2 ± 0.8	20.6 ± 0.2	359.2 ± 5.1 **	85.7 ± 0.5 **	6.3 ± 0.1	9.64	7.77	2.68	2.92
A4/RP1	83.0	106.3 ± 2.2 **	9.2 ± 0.8	19.5 ± 0.2 **	349.6 ± 4.0 **	77.7 ± 1.9 **	5.3 ± 0.1 **	8.32	7.35	2.47	3
A1/RP2	100.0	115.9 ± 2.3 **	8.8 ± 0.8	22.8 ± 0.2 **	298.4 ± 7.6 **	74.5 ± 0.9 **	5.1 ± 0.2 **	8.78	7.71	2.76	2.82
A2/RP2	104.0	105.8 ± 1.1 **	6.8 ± 0.8 *	23.9 ± 0.2 **	326.8 ± 9.3	76.9 ± 1.1 **	6.0 ± 0.2	8.96	8.26	2.75	3.02
A3/RP2	100.0	107.7 ± 1.2 **	9.0 ± 1.0	23.9 ± 0.1 **	267.4 ± 4.4 **	84.9 ± 0.9 **	5.4 ± 0.1 **	9.33	8.06	2.65	3.07
A4/RP2	84.0	104.3 ± 1.3 **	8.2 ± 0.8	22.3 ± 0.2 **	343.0 ± 3.6 **	75.0 ± 1.1.0 **	5.7 ± 0.1 *	8.87	7.7	2.52	3.07
A1/RP3	98.0	110.0 ± 3.5 **	10.2 ± 0.8 **	22.9 ± 0.1 **	298.4 ± 7.4 **	82.4 ± 0.6 **	5.6 ± 0.1 **	8.87	7.83	2.65	2.97
A2/RP3	103.0	101.3 ± 2.3 **	8.2 ± 0.8	22.6 ± 0.2 **	272.8 ± 12 **	82.8 ± 1.1 **	5.1 ± 0.1 **	8.23	8.23	2.73	3.03
A3/RP3	100.0	115.8 ± 2.5 **	9.6 ± 1.1	23.6 ± 0.1 **	227.4 ± 13.9 **	87.8 ± 1.5 *	4.7 ± 0.2 **	8.78	7.78	2.77	2.84
A4/RP3	85.0	105.3 ± 2.4 **	9.2 ± 0.8	21.1 ± 0.2	326.7 ± 15	81.2 ± 0.2 **	5.6 ± 0.2 *	8.32	7.67	2.52	3.06
A1/RP4	98.0	114.7 ± 1.0 **	8.4 ± 0.9	23.3 ± 0.2 **	356.1 ± 5.5 **	79.5 ± 0.9 **	6.6 ± 0.1 **	10.21	7.74	2.63	2.96
A2/RP4	103.0	102.8 ± 2.0 **	7.4 ± 0.9	21.4 ± 0.2 **	270.4 ± 3.1 **	72.4 ± 1.2 **	4.2 ± 0.1 **	8.32	8.18	2.63	3.13
A3/RP4	101.0	113.2 ± 2.5 **	9.2 ± 0.8	23.1 ± 0.1 **	244.3 ± 7.1 **	85.4 ± 0.2 **	4.8 ± 0.2 **	8.96	8.11	2.62	3.12
A4/RP4	84.0	105.9 ± 1.6 **	8.2 ± 0.8	20.5 ± 0.1	371.1 ± 11.8 **	85.4 ± 0.8 **	6.5 ± 0.1 *	9.67	7.62	2.49	3.09
A1/RP5	100.0	113.0 ± 1.3 **	7.8 ± 0.8	21.8 ± 0.1 **	336.4 ± 14.5	80.7 ± 0.7 **	5.9 ± 0.2	8.41	7.58	2.68	2.85
A2/RP5	106.0	119.5 ± 1.8	8.4 ± 0.9	21.7 ± 0.3 **	325.8 ± 9.6	74.7 ± 1.0 **	5.3 ± 0.1 **	7.10	7.67	2.71	2.85
A3/RP5	99.0	113.0 ± 3.3 **	8.0 ± 0.7	21.9 ± 0.3 **	222.8 ± 2.7 **	78.3 ± 1.6 **	3.8 ± 0.1 **	7.90	7.71	2.68	2.9
A4/RP5	86.0	111.3 ± 1.3 **	7.8 ± 0.8	23.4 ± 0.3 **	330.7 ± 6.2	72.8 ± 1.3 **	5.6 ± 0.2 *	8.59	8.2	2.63	3.14
Yongyou 1540	105.0	121.3 ± 2.0	8.2 ± 0.8	20.8 ± 0.2	321.2 ± 5.4	91.2 ± 1.2	6.1 ± 0.1	9.40	7.44	2.63	2.85

The agronomic traits were analyzed via *t*-test with Yongyou 1540 as the control (* indicates *p* < 0.05, ** indicates *p* < 0.01).

## Data Availability

The original contributions presented in this study are included in the article. Further inquiries can be directed to the corresponding author.
